# Role of riboflavin deficiency in malaria pathophysiology

**DOI:** 10.1371/journal.ppat.1011991

**Published:** 2024-03-01

**Authors:** Shweta Sharma, Arathy Ramachandran, Amit Sharma

**Affiliations:** 1 ICMR–National Institute of Malaria Research, New Delhi, India; 2 ACSIR, Ghaziabad, Uttar Pradesh, India; 3 Molecular Medicine Group, International Centre for Genetic Engineering and Biotechnology, New Delhi, India; University of Wisconsin Medical School, UNITED STATES

## Abstract

The emergence of resistance against antimalarials and insecticides poses a significant threat to malaria elimination strategies. It is crucial to explore potential risk factors for malaria to identify new targets and alternative therapies. Malnutrition is a well-established risk factor for malaria. Deficiencies of micronutrients such as vitamin A, zinc, iron, folic acid, and phenotypic measures of malnutrition, such as stunting and wasting, have been studied extensively in the context of malaria. Vitamin B2, also known as riboflavin, is a micronutrient involved in maintaining cellular homeostasis. Riboflavin deficiency has been shown to have an inverse correlation with malarial parasitaemia. This article reviews the role of riboflavin in maintaining redox homeostasis and probes how riboflavin deficiency could alter malaria pathogenesis by disrupting the balance between oxidants and antioxidants. Though riboflavin analogues have been explored as antimalarials, new *in vivo* and patient-based research is required to target riboflavin-associated pathways for antimalarial therapy.

## Background

Malaria is a vector-borne disease caused by the parasites of *Plasmodium* species and transmitted through the bites of female *Anopheles* mosquitoes [1]. It remains a significant public health concern in countries in sub-Saharan Africa, south and south-east Asia, the Middle East, South America, and Oceania. Even though the number of deaths due to malaria has significantly reduced over the past 2 decades, the decline in malaria incidence has plateaued. The global malaria incidence was estimated to be 245 million in 2000 and 249 million in 2022 [2]. Global spending on malaria has only increased since 2000 and was estimated to be more than $ 4.3 billion in 2016 [3]. Diverse factors have contributed to the fall in the rate of decline of malaria, such as the emergence of resistance against drugs and insecticides, invasive vector species, poor epidemiological and vector surveillance, and poor allocation of resources [4]. Surveillance and resource allocation are interdependent and mutually reinforcing and help identify risk factors associated with malaria in that specific population.

Nutritional status is a complex risk factor affecting malaria incidence and treatment. There is substantial evidence that malaria incidence is associated with stunting, wasting, protein-energy malnutrition, and micronutrient deficiencies in humans [5,6]. Understanding how nutrient deficiency modulates malaria pathology will help discover novel drug targets and design adjuvant therapeutic strategies. It will also aid in identifying vulnerable populations and improve disease management. One such micronutrient is vitamin B2 or riboflavin. Free riboflavin is metabolised into FMN and FAD, which act as cofactors for glutathione reductase (GR). Therefore, riboflavin deficiency can lead to reduced GR activity and hence, redox imbalance in cells and ultimately cell death. It has been hypothesised that riboflavin deficiency lowers malarial parasitaemia through oxidative stress-induced death of parasite infected RBCs. However, *in vitro* and *in vivo* studies have demonstrated that riboflavin deficiency is associated with reduced parasitaemia. Contradictory reports and lack of evidence from human subjects weaken these conclusions [7]. In this “Pearls” article, we discuss the role of riboflavin signalling in the maintenance of the redox status of cells, its dysfunction during *Plasmodium* infection, the influence of host genetics, and its potential as an antimalarial target.

### How does riboflavin help maintain the balance between oxidants and antioxidants?

**Fig 1:**
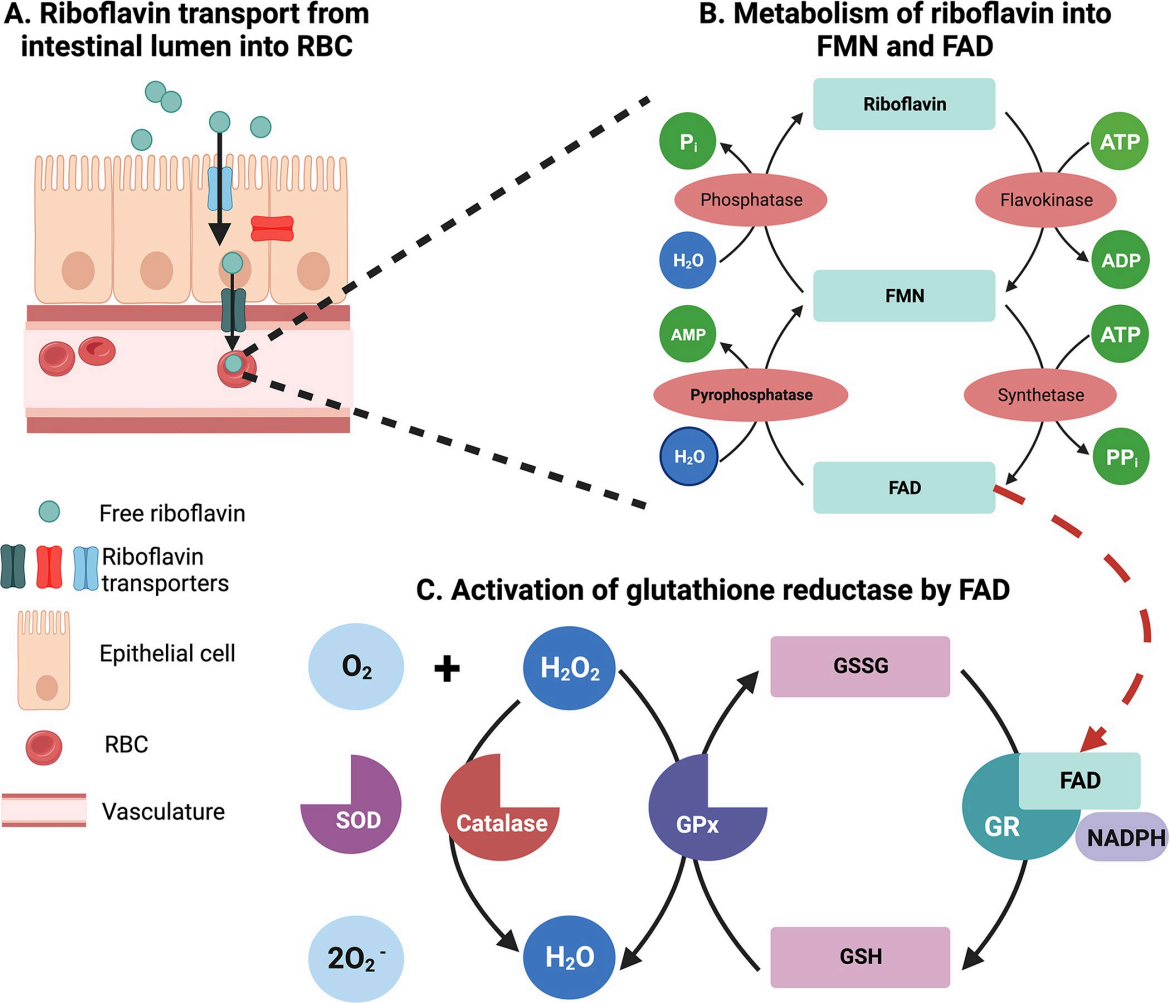
Role of riboflavin in the maintenance of cellular homeostasis. Riboflavin, also known as vitamin B2, has to be sourced externally by the mammalian cells from food rich in vitamin B2, such as milk and milk products, eggs, lean meat, salmon, almonds, and spinach. The Recommended Dietary Allowance for men and women is 1.3 mg and 1.1 mg daily, respectively. Once in the intestinal lumen, it is absorbed into the gut epithelial cells (brush border) through riboflavin transporters (hRFT). There are 3 known riboflavin transporters: hRFT1, hRFT2, and hRFT3. The specific function and anatomical distribution of each transporter is unclear. However, it is believed that hRFT3 helps translocate riboflavin from the lumen into the gut epithelium, and hRFT1 and hRFT2 help transport it to the RBCs. Once inside the RBC, free riboflavin is sequentially metabolised by flavokinase and FAD synthetase to generate FMN and FAD. FAD acts as a coenzyme for GR and helps transfer electrons from NADPH to oxidised glutathione disulfide (GSSG), thus generating reduced glutathione (GSH). GSH helps maintain redox homeostasis by reducing biomolecules such as sulfhydryl groups. Superoxide dismutase converts superoxide ions into oxygen and hydrogen peroxide (H_2_O_2_). H_2_O_2_ is neutralised into H_2_O and O_2_ by catalase and glutathione peroxidase (GPx). GPx, in turn, is dependent on GSH for this neutralisation. Therefore, riboflavin is a crucial member of this molecular network that maintains a cell’s balance between oxidants and antioxidants.

Riboflavin is an essential micronutrient crucial for cellular homeostasis. It is sourced exogenously and absorbed into the blood through the intestine. There are 3 known human riboflavin transporters (hRFT1, hRFT2, and hRFT3). The hRFT1 and hRFT2 are expressed on the basal and lateral membrane of the gut epithelial cell, while hRFT3 is expressed on the brush border [8]. The expression pattern of the transporters suggests that hRFT3 facilitates the transport of free riboflavin from the lumen into the epithelial cell, while hRFT1 and hRFT2 are involved in the uptake of riboflavin from the epithelial cells into red blood cells (RBCs) [9]. Free riboflavin in the cells is phosphorylated to generate flavin mononucleotide (FMN), which is then further metabolised to flavin adenine dinucleotide (FAD). FMN and FAD are cofactors for other enzymes, including GR. FAD binds with GR and transfers the H+ from NADPH to glutathione disulphide (oxidised glutathione—GSSG). This electron transfer reduces GSSG to glutathione (GSH) [10]. Riboflavin derivatives (FMN and FAD) along with other cofactors (copper and zinc) and enzymes (GR, superoxide dismutase, glutathione peroxidase, and catalase) balance the redox status of a cell (Fig 1). Therefore, riboflavin deficiency can lead to oxidative stress through an increase in the levels of cellular reactive oxygen species. Oxidative stress can further trigger the activation of cellular pathways, such as inflammation and apoptosis, which is detrimental to the cell’s survival. Chronic riboflavin deficiency has been implicated as a risk factor for cardiovascular disease, cancer, anaemia, neurodegeneration, and infectious diseases [11]. However, its role in the pathogenesis of malaria remains ambiguous.

### How does riboflavin deficiency affect malaria pathogenesis?

**Fig 2:**
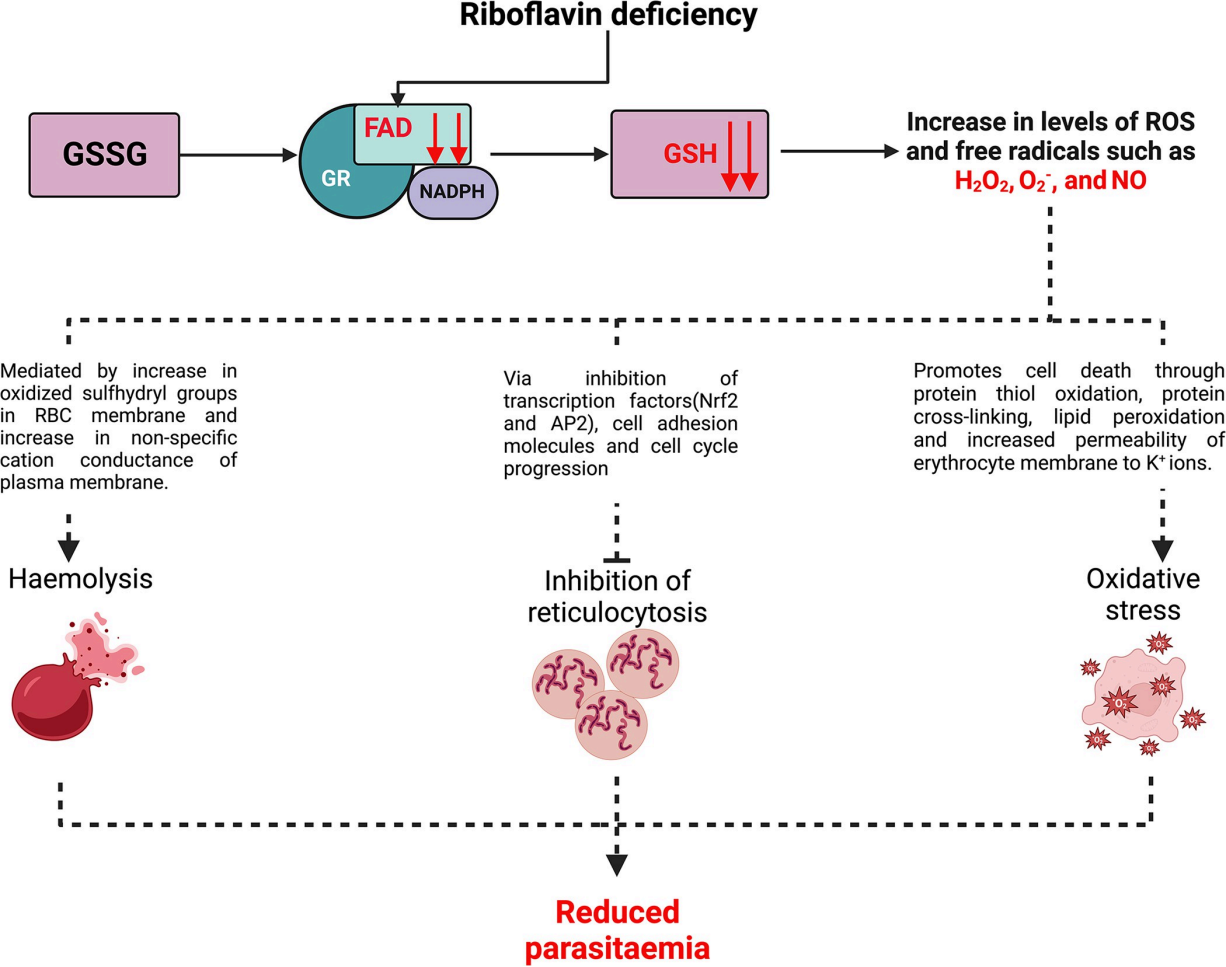
Impaired riboflavin signalling and its role in the pathogenesis of malaria. Riboflavin deficiency will lead to reduced levels of free riboflavin in the RBCs. This will result in low levels of FMN and FAD. Since GR is dependent on FAD for reducing GSSG into GSH, low levels of FAD will inhibit GR activity and, hence, lead to reduced levels of GSH in the cell. GSH is a crucial antioxidant; low GSH levels will increase free radicals and ROS, such as superoxide ions, H_2_O_2,_ and nitric oxide. Three main hypotheses were proposed to explain how riboflavin deficiency can lead to low parasitaemia: haemolysis, inhibition of reticulocytosis, and oxidative stress. Sulfhydryl groups form an essential part of the erythrocyte membrane. Low levels of antioxidants in a cell lead to an increase in oxidised membrane sulfhydryl groups, negatively impacting membrane integrity. An increase in ROS levels has also been shown to increase nonspecific cation conductance of the plasma membrane, thus affecting the osmotic balance of the cell. High levels of ROS have also been shown to alter the expression patterns of several housekeeping genes, such as FOX3A, NF kappa B, and AP-2. These proteins act as transcription factors for other genes, such as ICAM1 and TNF alpha which are essential for generating new RBCs (reticulocytosis). ROS have also been shown to interfere with erythropoietic stem cell (ESC) cell division. Therefore, increased levels of ROS and free radicals in ESCc will affect reticulocytosis. Similarly, free radicals and ROS can promote programmed cell death or apoptosis through redox modification of proteins, carbohydrates, lipids, and nucleic acids. These mechanisms can trigger cell death either in synergy or in isolation. While cell death (loss of RBCs) under normal conditions can lead to anaemia, loss of infected RBCs can lead to reduced parasitaemia and protection against malaria. However, it is unclear if the protective effect shown by riboflavin deficiency outweighs the significant haemoglobin or RBC loss associated with riboflavin deficiency-induced anaemia.

The association between riboflavin status and parasite density has been studied since the 1940s. Multiple *in vitro* studies demonstrated the inverse correlation between parasitaemia and riboflavin deficiency, i.e., riboflavin deficiency lowers parasite count [12]. These findings contradicted the long-withstanding belief that malnutrition makes one more susceptible to infections. Population-based studies in Papua New Guinea and Gambia showed that infants in these regions had low riboflavin levels, and malaria incidence was lower in riboflavin-deficient individuals [13]. Similarly, another study from Orissa, India reported that the malarial parasitaemia was low in individuals with low riboflavin levels [14]. Three hypotheses were put forth to explain how riboflavin deficiency leads to low parasitaemia: (i) through increasing the susceptibility of RBCs to haemolysis; (ii) through inhibition of reticulocytosis; and (iii) through generating oxidative stress in the iRBCs (Fig 2).

Haemolysis and oxidative stress are not mutually exclusive of each other. Some molecular regulators are shared between the 2 pathways, such as GSH and glutathione peroxidase (GPx). GSH deactivates cellular peroxides with the help of GPx. Riboflavin deficiency can lead to low levels of FMN and FAD and, hence, low levels of GSH. A decrease in GSH levels will down-regulate GPx activity and lead to an increase in lipid peroxidation, which ultimately will cause oxidative stress [15]. GSH and GPx are also crucial for the oxidation of sulfhydryl groups. The sulfhydryl groups play a significant role in maintaining the structural integrity of the RBC membrane, and an imbalance in their redox status can lead to haemolysis [16]. Similarly, GSSG accumulation in cells can dysregulate hexose monophosphate pathway, affecting the cell’s energy supply. These mechanisms, individually or in synergy, will lead to cell death and, hence, will reduce the number of RBCs, lowering the number of iRBCs. FMN and FAD also act as cofactors for heme synthesis. Riboflavin deficiency can lower heme synthesis and down-regulate reticulocytosis [17]. Fewer reticulocytosis could mean lesser RBCs for the parasites to infect and, hence, lower parasitaemia. *P*. *falciparum* does not have genes for enzymes involved in riboflavin biosynthesis and the literature on whether the parasite requires an external supply of riboflavin is conflicting [18]. Putative flavokinase and FAD synthetase enzymes (MAL13P1.292 and Pf10_0147) have been identified in *P*. *falciparum* but it is unclear how riboflavin is transported and metabolised in the parasite [19]. There is no conclusive evidence that human riboflavin deficiency affects either one’s susceptibility to or alters the prognosis of malaria. A lesser explored aspect that would further our understanding of the association between riboflavin and malaria is whether riboflavin deficiency has been selected for genetically in individuals living in malaria-endemic regions.

### Is there an evolutionary selective pressure on riboflavin deficiency in the context of malaria?

Riboflavin deficiency has been reported in newborns and infants in Papua New Guinea and Gambia [13]. This deficiency has also been shown to be inversely related to malaria incidence. These reports support the hypothesis that severe riboflavin deficiency at birth in these countries might be a coevolved adaptation for malaria and riboflavin. Similarly, native Amazonians (with or without malaria) have reduced cellular GSH activity compared to the migrants living in the same region [20]. The low GSH activity could be due to low riboflavin levels or down-regulation of riboflavin metabolism. *In vitro* studies reported that under oxidative stress conditions, parasite growth is significantly inhibited in RBCs derived from individuals carrying thalassemia and glucose-6-phosphate dehydrogenase (G6PD) mutations [21]. In line with this, higher incidence and severity of FAD/FMN deficiency were observed in individuals homozygous for thalassemia mutation and their heterozygous relatives than in their genetically unrelated spouses [22]. Also, the low cellular FAD/FMN levels were independent of peripheral riboflavin levels [22]. It is unclear how the intracellular levels of FAD/FMN are regulated and how it is associated with thalassemia and G6PD mutations. Single-nucleotide polymorphisms in riboflavin transporters, which could affect intracellular levels of riboflavin, have been reported, but their association with malaria has not been studied yet [23]. In conclusion, although multiple studies have provided indirect evidence that riboflavin metabolism and levels could be genetically regulated, it is unclear how these regulations are associated with malaria [24–26]. Though the mechanistic pathways remain poorly understood, riboflavin has been considered a potential antimalarial target since the 1940s.

### Can the riboflavin metabolism pathway be targeted for antimalarial therapy?

Since riboflavin deficiency has demonstrated antiparasitic activity *in vitro* and *in vivo*, 2 kinds of drugs have been studied for antimalarial activity: riboflavin analogues and riboflavin deficiency-inducing drugs. The analogues are mostly a series of 10-(4′-chlorophenyl)-3-substituted flavin compounds. The *in vitro* activity of these compounds has been tested on *Plasmodium falciparum*, while the *in vivo* activity has been tested on mice infected with *Plasmodium vinckei vinckei* [27]. Two compounds, 10-(4′-chlorophenyl)-3-methylflavin and roseoflavin (IC_50_ = 120 nM), demonstrated significant antiparasitic activity in both cultured infected RBCs and mice models of malaria [27,28]. The riboflavin deficiency inducing (RDI) drug chlorpromazine has shown significant *in vitro* and *in vivo* antimalarial activity through the simulation of eryptosis [29]. However, further clinical studies are required to determine its efficiency against human malaria. Another similar RDI compound imipramine is an efficient chloroquine resistance-reversing agent *in vitro* but has not yet been used against human malaria due to poor bioavailability and its lack of metabolic stability [30].

Despite such exciting findings, there has yet to be any success in discovering new antimalarials or adjuvant therapies targeting riboflavin deficiency or metabolism. There are 3 main reasons for this. First, there are gaps in our understanding of how riboflavin signalling is modulated in malaria and how riboflavin analogues lower parasitaemia. Second, not all studies found a significant correlation between riboflavin deficiency and parasitaemia. Riboflavin supplementation in Gambian children was shown to have no effects on malaria incidence [31]. A lower riboflavin concentration was observed in Nigerian children with *P*. *falciparum* malaria compared to healthy controls, which contradicts the earlier studies reporting a lower incidence of malaria in riboflavin-deficient children [32]. It was also reported that in most *in vivo* models, where malaria and riboflavin deficiency were introduced, significantly higher mortality was observed in the riboflavin-deficient animals compared to the control animals [7]. Another significant concern is that the haemolysis induced by riboflavin deficiency might contribute to malaria-associated anaemia, aggravating the disease [5]. Lastly, there are limitations associated with mouse models of malaria as they do not exhibit all aspects of human infection and disease [33]. In such a scenario, it is challenging to interpret and translate the experimental results to design new drugs.

In summary, sparse conclusive evidence exists to suggest that riboflavin deficiency lowers parasitaemia or malaria incidence in humans. Therefore, *in vitro* experiments such as exposing *P*. *falciparum* cultures to riboflavin-deficient media and comparing their viability to those in riboflavin-supplemented media, studying the effects of different concentrations of riboflavin on parasite growth and replication, and investigating the impact of riboflavin on mitochondrial function of *P*. *falciparum* such as membrane potential, ATP production and ROS generation will further our understanding of the role of riboflavin deficiency in malaria pathology. Additionally, there is evidence suggesting that riboflavin levels and metabolism are influenced by host genetics, but the mechanisms remain to be understood. Investigating population-specific association between riboflavin deficiency, host genetic polymorphisms in riboflavin transporters, and malaria incidence will provide deeper insights on possible selective pressures imposed by malaria on riboflavin metabolism in a population. Studies to enhance the bioavailability and metabolic stability of existing riboflavin analogues and RDI drugs will be crucial for translating these compounds from *in vitro* and *in vivo* studies to possible clinical use. This will also help identify new targets for antimalarial therapy in the riboflavin deficiency–oxidative stress signalling axis. With an increase in global incidence of malaria over past 2 years and spread of antimalarial resistance, it is imperative that we explore these alternative avenues for developing effective antimalarials.
